# Supplementary material for: Venomix: a simple bioinformatic pipeline for identifying and characterizing toxin gene candidates from transcriptomic data
Source: PeerJ. 2018 Jul 31;6:e5361. doi: 10.7717/peerj.5361 (PMC6074769; doi:10.7717/peerj.5361)
Supplement: Supplemental Information 3 [file peerj-06-5361-s003.gz › FinalOutput_E-6/Phospholipase_A2_homolog_crotoxin_acid_subunit_CA_21/finaltree.pdf]

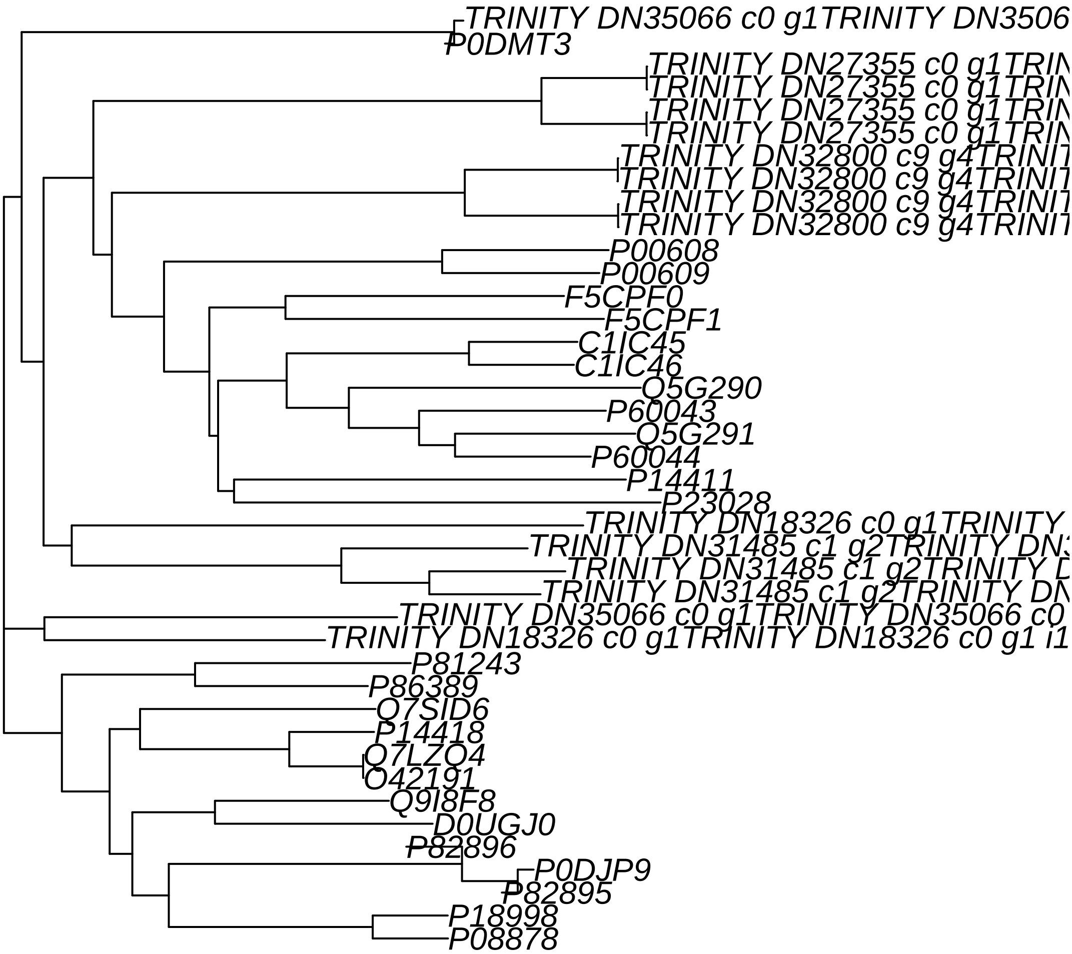
TRINITY DN35066 c0 g1TRINITY DN3506
P0DMT3
TRINITY DN27355 c0 g1TRIN
TRINITY DN27355 c0 g1TRIN
TRINITY DN27355 c0 g1TRIN
TRINITY DN27355 c0 g1TRIN
TRINITY DN32800 c9 g4TRINIT
TRINITY DN32800 c9 g4TRINIT
TRINITY DN32800 c9 g4TRINIT
TRINITY DN32800 c9 g4TRINIT
P00608
P00609
F5CPF0
F5CPF1
C1IC45
C1IC46
Q5G290
P60043
Q5G291
P60044
P14411
P23028
TRINITY DN18326 c0 g1TRINITY
TRINITY DN31485 c1 g2TRINITY DN
TRINITY DN31485 c1 g2TRINITY D
TRINITY DN31485 c1 g2TRINITY DN
TRINITY DN35066 c0 g1TRINITY DN35066 c0
TRINITY DN18326 c0 g1TRINITY DN18326 c0 g1 i1
P81243
P86389
Q7SID6
P14418
Q7LZQ4
O42191
Q9I8F8
D0UGJ0
P82896
P0DJP9
P82895
P18998
P08878
